# Anti-Inflammatory Effects of Diospyrin on Lipopolysaccharide-Induced Inflammation Using RAW 264.7 Mouse Macrophages

**DOI:** 10.3390/biomedicines8010011

**Published:** 2020-01-11

**Authors:** Adnan Shahidullah, Ji-Young Lee, Young-Jin Kim, Syed Muhammad Ashhad Halimi, Abdur Rauf, Hyun-Ju Kim, Bong-Youn Kim, Wansu Park

**Affiliations:** 1 Department of Pharmacy, University of Peshawar, Peshawar 25120, Pakistan; adnansuk1@gmail.com (A.S.); ashhad92@gmail.com (S.M.A.H.); 2 College of Korean Medicine, Gachon University, Seong-nam 13120, Korea; oxygen1119@naver.com (J.-Y.L.); godsentry@naver.com (Y.-J.K.); eternity0304@daum.net (H.-J.K.); famous008@daum.net (B.-Y.K.); 3 Department of Chemistry, University of Swabi, Anbar 23561, KPK, Pakistan; mashaljcs@yahoo.com

**Keywords:** diospyrin, lipopolysaccharide, anti-inflammation, macrophages, nitric oxide, cytokine, calcium, CHOP, Fas, p38 MAPK

## Abstract

Diospyrin is a bisnaphthoquinonoid medicinal compound derived from *Diospyros lotus*, with known anti-cancer, anti-tubercular, and anti-leishmanial activities against *Leishmania donovani*. However, the effects of diospyrin on lipopolysaccharide (LPS)-induced macrophage activation and inflammation are not fully reported. In this study, the anti-inflammatory effects of diospyrin on LPS-induced macrophages were examined. Diospyrin showed no toxicity in RAW 264.7 at concentrations of up to 10 μM. Diospyrin moderated the production of nitric oxide (NO), monocyte chemotactic protein-1, macrophage inflammatory protein-1β, interleukin (IL)-6, IL-10, granulocyte colony-stimulating factor, granulocyte macrophage colony-stimulating factor, vascular endothelial growth factor, leukemia inhibitory factor, and RANTES/CCL5, as well as calcium release in LPS-induced RAW 264.7, at concentrations of up to 10 μM significantly (*p* < 0.05). Diospyrin also significantly inhibited the phosphorylation of p38 mitogen-activated protein kinase (MAPK) and mRNA expression of C/EBP homologous protein (CHOP), as well as tumor necrosis factor receptor superfamily member 6 (Fas), in LPS-induced RAW 264.7 cells at concentrations of up to 10 μM (*p* < 0.05). Diospyrin exhibits anti-inflammatory properties mediated via inhibition of NO, and cytokines in LPS-induced mouse macrophages via the ER-stressed calcium-p38 MAPK/CHOP/Fas pathway.

## 1. Introduction

Immunity is essential for life. Innate immunity is important in protecting the host against the invasion of pathogenic microorganisms, including viruses, bacteria, fungi, and parasitic protozoa, as well as preventing tumor occurrence. Capone et al. reported that inflammation is an immune and physiological event in response to infection and/or several types of trauma [1]. Warnatsch et al. reported that inflammation is critical against infection, but must be regulated to prevent inflammatory disease [2].

Macrophage is a representative phagocyte. Macrophages are important inflammatory cells implicated in the initiation of inflammatory responses. They play a critical role in the pathogenesis of numerous inflammatory diseases by secreting various pro-inflammatory mediators and pro-inflammatory cytokines [3].

Zhang et al. reported that lipopolysaccharide (LPS) is regarded to be a key factor in the pathogenesis of bacterial sepsis [4].

Cytokine storm is thought to be important in the uncontrolled inflammation, which could be provoked by serious bacterial infections. Till now, treatments for cytokine storm are insufficient in spite of various antibiotics and many new vaccines. Natural compounds sometimes receive attention owing to their nontoxicity and anti-inflammatory activities. With anti-inflammatory effects, the signaling pathway of natural compounds for infections deserves careful study.

**Figure 1:**
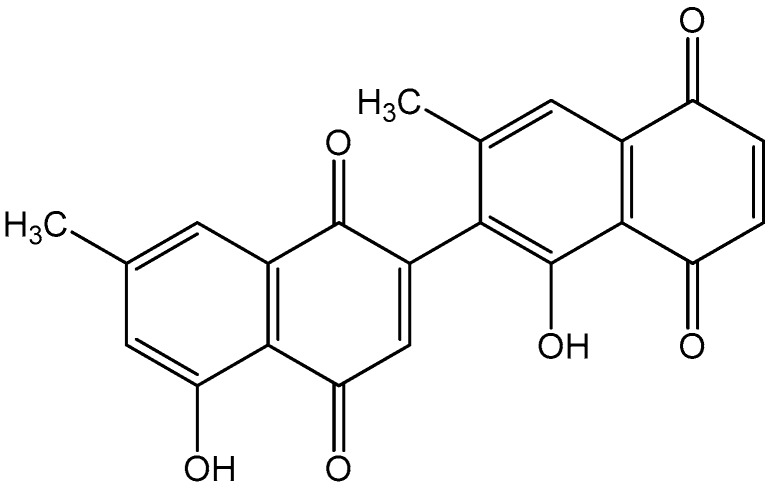
Chemical structure of diospyrin.

Diospyrin (Figure 1), the bisnaphthoquinonoid product derived from the medicinal plant *Diospyros lotus*, is known to exhibit anti-cancer, anti-tubercular, and anti-leishmanial activity against *Leishmania donovani* [5]. However, the effects of diospyrin on LPS-induced macrophages are not fully reported.

In the present study, we investigated the inhibitory effects of diospyrin on LPS-induced inflammation using RAW 264.7 mouse macrophages. Treatment with diospyrin concentrations up to 10 μM significantly modulated the excessive production of inflammatory mediators such as nitric oxide (NO), cytokines, chemokines, and growth factors. It also inhibited calcium release, phosphorylation of p38 mitogen-activated protein kinase (MAPK), and mRNA expression of C/EBP homologous protein (CHOP) and tumor necrosis factor receptor superfamily member 6 (Fas) in LPS-induced RAW 264.7 cells.

## 2. Materials and Methods

### 2.1. Materials

Dulbecco’s modified Eagle’s medium (DMEM), FBS, penicillin, streptomycin, PBS, and other cell culture reagents were purchased from Millipore (Billerica, MA, USA). Diospyrin was isolated from *Diospyros lotus* by Dr. Inamullah Khan. Multiplex cytokine assay kits were purchased from Millipore. The Fluo-4 calcium assay kit was supplied by Molecular Probes (Eugene, OR, USA). Real-time RT-PCR kits were ordered from Bio-Rad (Hercules, CA, USA). Phospho-p38 MAPK Antibody (T180/Y182) (eBioscience 17-9078-42) and Mouse IgG2b kappa Isotype Control (eBioscience 12-4732-81) were obtained from Life Technologies Corporation (Carlsbad, CA, USA). All other solutions for flow cytometric analysis were purchased from Thermo Fisher Scientific (Waltham, MA, USA).

### 2.2. Cell Viability Assay

RAW 264.7 cell line were obtained from Korea Cell Line Bank (Seoul, Korea). The modified MTT assay was used to evaluate the effect of diospyrin on viability of RAW 264.7 [6].

### 2.3. Quantification of NO Production

Cell were incubated with compounds for 24 h in 96-well plates and NO level in each well was evaluated using the Griess reagent assay kit (Millipore) according the manufacturer’s protocol at 540 nm with a microplate reader (Bio-Rad) [6]. Indomethacin (0.5 µM) was used as a positive control.

### 2.4. Intracellular Calcium Assay

Cell were incubated with compounds for 18 h in 96-well plates and the intracellular calcium signaling from each well was evaluated using Fluo-4 NW Calcium Assay Kits (Thermo Fisher Scientific) according the manufacturer’s protocol by a spectrofluorometer (Dynex, West Sussex, UK), with excitation and emission filters of 485 nm and 535 nm [7]. Indomethacin (0.5 µM) was used as a positive control.

### 2.5. Cytokines Production

Cell were incubated with materials for 24 h in 96-well plates and productions of various cytokines from each well were evaluated using Multiplex cytokine assay kits (Millipore) and Bio-Plex 200 suspension array system (Bio-Rad) according the manufacturer’s protocols [7,8].

### 2.6. RNA Isolation and Real Time RT-PCR Analysis

Cell were incubated with materials for 18 h in six-well plates and RNA quantity was evaluated with real time RT-PCR analysis using NucleoSpin RNA kit (Macherey-Nagel, Duren, Germany), iScript cDNA Synthesis kit (Bio-Rad), the Experion RNA StdSens Analysis kit (Bio-Rad), iQ SYBR Green Supermix (Bio-Rad), and Experion Automatic Electrophoresis System (Bio-Rad) [8,9]. The target genes are listed in Table 1. The *β*-actin was used as a reference.

### 2.7. Flow Cytometry

After 15 min of incubation in six-well plates with compounds, cells were harvested and washed with a flow cytometry staining buffer (Thermo Fisher Scientific, Waltham, MA, USA). The cells were stained with anti-phospho-p38 MAPK antibodies according to the manufacturer’s protocol. Prior to antibody staining, cells were fixed and permeabilized using Fix Buffer I (Thermo Fisher Scientific) and Perm Buffer III (Thermo Fisher Scientific), respectively. Stained cells were analyzed on the Attune NxT flow cytometer (Thermo Fisher Scientific). Unstained cells were used as negative gating control. Mouse IgG2b kappa Isotype Control was used to confirm the specificity of ohospho-p38 MAPK antibody. The raw data were analyzed using Attune NxT software (Thermo Fisher Scientific). Baicalein (25 µM) was used as a positive control. In this assay, the concentration of LPS was 0.1 µg/mL because LPS at the concentration of 1 µg/mL caused excessive effects on cellular reaction, which made the evaluation of the signaling pathway difficult.

### 2.8. Statistical Analysis

Data are presented as mean ± SD. All data were analyzed by one-way analysis of variance test using GraphPad Prism (ver. 4; GraphPad Software, San Diego, CA, USA).

## 3. Results

### 3.1. Effect of Diospyrin on Cell Viability

**Figure 2:**
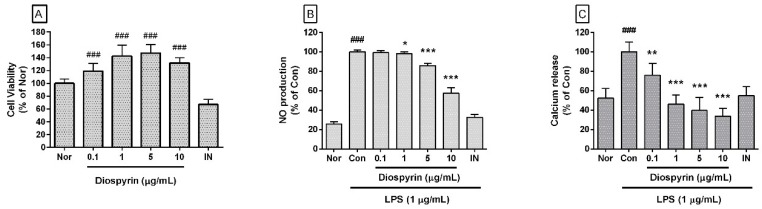
Effect of diospyrin on cell viability, NO production, and calcium release in lipopolysaccharide (LPS)-induced RAW 264.7 cells. After 24 h treatment, cell viability (**A**) was evaluated via a modified MTT assay and nitric oxide (NO) production (**B**) was measured using the Griess reaction assay. Calcium release (**C**) was measured with Fluo-4 calcium assay after 18 h treatment. Data are of mean ± SD and pooled from more than three independent experiments. Nor, normal group (media only); Con, control group (LPS alone); IN, indomethacin (0.5 µM). ^###^ *p* < 0.001 vs. Nor; * *p* < 0.05 vs. Con; ** *p* < 0.01 vs. Con; *** *p* < 0.001 vs. Con.

In this study, diospyrin up to a concentration of 10 µM restored the viability of RAW 264.7 cells (Figure 2A). The cell viabilities of RAW 264.7 cells, which were incubated with diospyrin at concentrations of 0.1, 1, 5, and 10 µM/mL for 24 h were 118.84% ± 11.87%, 142.4% ± 17.47%, 147.22% ± 13.28%, and 131.65% ± 8.51%, respectively, of the normal group treated with media only. On the basis of this result, diospyrin concentrations of up to 10 µM were selected for use in subsequent experiments.

### 3.2. Effect of Diospyrin on NO Production

Diospyrin significantly inhibited the excessive production of NO in LPS-induced RAW 264.7 cells (Figure 2B). Percentages of NO production in LPS-induced RAW 264.7 cells incubated with diospyrin at concentrations of 0.1, 1, 5, and 10 μM for 24 h were 99.15% ± 2.12%, 97.94% ± 2.11%, 85.76% ± 2.5%, and 57.35% ± 5.74%, respectively, of the control group treated with LPS only.

### 3.3. Effect of Diospyrin on Intracellular Calcium Release

In the present study, diospyrin significantly inhibited the calcium release in LPS-induced RAW 264.7 cells (Figure 2C). Percentages of calcium release in LPS-induced RAW 264.7 cells incubated with diospyrin at concentrations of 0.1, 1, 5, and 10 μM for 18 h were 75.84% ± 11.98%, 46.06% ± 9.44%, 39.5% ± 13.49%, and 33.63% ± 8.18%, respectively, of the control group treated with LPS alone.

### 3.4. Effect of Diospyrin on Cytokine Production

**Figure 3:**
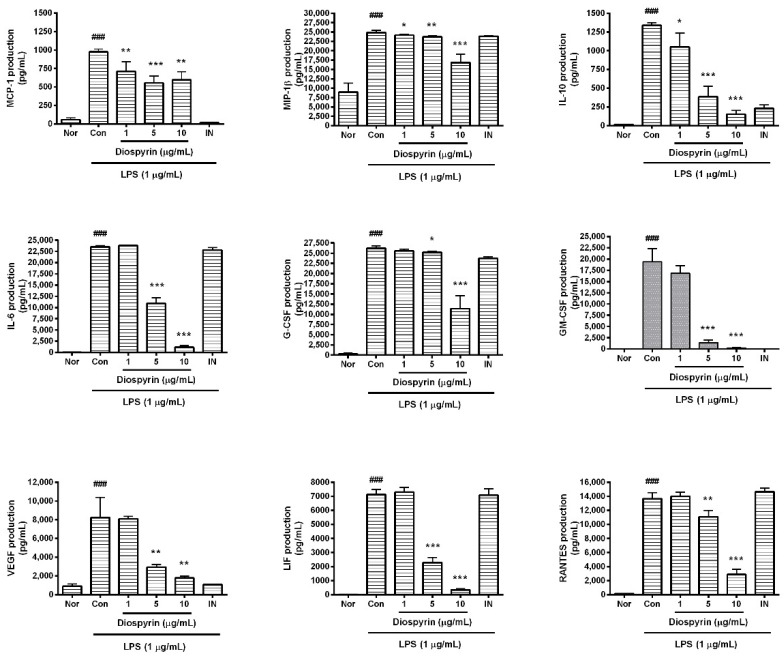
Effect of diospyrin on production of monocyte chemotactic protein (MCP)-1, macrophage inflammatory protein (MIP)-1β, granulocyte colony-stimulating factor (G-CSF), granulocyte macrophage colony-stimulating factor (GM-CSF), vascular endothelial growth factor (VEGF), RANTES/CCL5, leukemia inhibitory factor (LIF; IL-6 class cytokine), interleukin (IL)-6, and IL-10 in LPS-stimulated RAW 264.7. Data are of mean ± SD and pooled from more than three independent experiments. Nor, normal group (media only); Con, control group (LPS alone); IN, indomethacin (0.5 µM). ^###^ *p* < 0.001 vs. Nor; * *p* < 0.05 vs. Con; ** *p* < 0.01 vs. Con; *** *p* < 0.001 vs. Con.

In the present study, diospyrin significantly reduced the excessive synthesis of monocyte chemotactic protein (MCP)-1, macrophage inflammatory protein (MIP)-1β, granulocyte colony-stimulating factor (G-CSF), granulocyte macrophage colony-stimulating factor (GM-CSF), vascular endothelial growth factor (VEGF), RANTES/CCL5, leukemia inhibitory factor (LIF; IL-6 class cytokine), interleukin (IL)-6, and IL-10 in LPS-induced RAW 264.7 cells (Figure 3). In detail, the production of MCP-1 in LPS-induced RAW 264.7 cells incubated with diospyrin at concentrations of 1, 5, and 10 µM for 24 h were 73.02% ± 13.18%, 57.02% ± 9.51%, and 61.22% ± 11.23%, respectively, of the control group treated with LPS only. The levels of MIP-1β were 97.11% ± 0.84%, 95.24% ± 1.3%, and 67.74% ± 8.73%, respectively. The levels of IL-10 following treatment with the three different concentrations of diospyrin were 78.56% ± 13.8%, 28.81% ± 10.48%, and 11.32% ± 3.87%, respectively. The levels of IL-6, G-CSF, GM-CSF, VEGF, LIF, and RANTES, following exposure to the three different concentrations of diospyrin, were as follows: 100.94% ± 1.16%, 46.37% ± 5.6%, and 4.94% ± 1.56%; 97.55% ± 1.78%, 96.19% ± 1.04%, and 43.51% ± 12.16%; 86.56% ± 8.86%, 7.15% ± 2.96%, and 1.12% ± 0.5%; 98.28% ± 3.11%, 35.78% ± 3.16%, and 21.55% ± 2.64%; 102.46% ± 4.75%, 32.2% ± 5.12%, and 4.81% ± 1.13%; and 102.55% ± 4.47%, 81.13% ± 6.81%, and 21.08% ± 5.4%, respectively.

### 3.5. Effect of Diospyrin on mRNA Expression of CHOP and Fas

**Figure 4:**
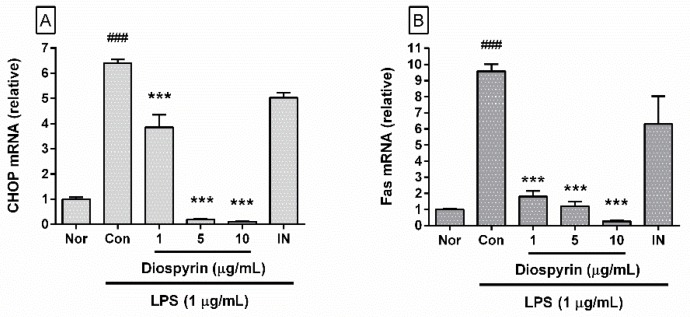
Effect of diospyrin on mRNA expression of C/EBP homologous protein (CHOP) (**A**) and Fas (**B**) in LPS-induced RAW 264.7 were measured with real-time RT-PCR after 18 h incubation with compounds. β-actin was used as the housekeeping gene. Nor, normal group (media only); Con, control group (LPS alone). IN denotes indomethacin (0.5 µM). Values are the mean ± SD of three independent experiments. ^###^ *p* < 0.001 vs. Nor; *** *p* < 0.001 vs. Con.

In the present study, diospyrin significantly inhibited mRNA expression of CHOP and Fas in LPS-induced RAW 264.7 cells (Figure 4). In detail, the levels of mRNA expression of CHOP in LPS-induced RAW 264.7 cells incubated with diospyrin at concentrations of 1, 5, and 10 µM for 18 h were 60.19% ± 7.79%, 3.1% ± 0.18%, and 1.71% ± 0.09%, respectively, of the control group treated with LPS only (Figure 4A), and the corresponding levels of Fas mRNA were 18.9% ± 3.54%, 12.69% ± 2.92%, and 2.86% ± 0.51%, respectively (Figure 4B). The data suggest that diospyrin inhibits calcium release from endoplasmic reticulum stores and the inflammatory reaction in LPS-induced mouse macrophages via calcium-CHOP/Fas pathway.

### 3.6. Effect of Diospyrin on Phosphorylation of p38 MAPK

**Figure 5:**
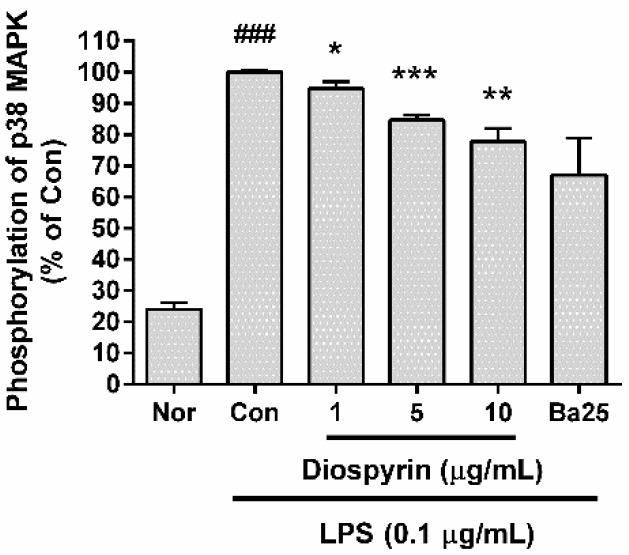
Effect of diospyrin on the phosphorylation of p38 MAPK in LPS-induced RAW 264.7 cells. After 15 min of treatment, the phosphorylation of p38 MAPK was measured via flow cytometry. The normal group (Nor) was treated with media only. The control group (Con) was treated with LPS (0.1 µg/mL) alone. Ba25 denotes baicalein (25 µM). Values are the mean ± SD of three independent experiments. ^###^ *p* < 0.001 vs. Nor; * *p* < 0.05 vs. Con; ** *p* < 0.01 vs. Con; *** *p* < 0.001 vs. Con.

In the present study, diospyrin significantly inhibited the phosphorylation of p38 MAPK in LPS-induced RAW 264.7 cells (Figure 5). The phosphorylation of p38 MAPK in LPS-induced RAW 264.7 cells incubated with diospyrin at concentrations of 1, 5, and 10 µM for 15 min were 94.76% ± 2.14%, 84.67% ± 1.66%, and 77.77% ± 4.2%, respectively of the control group treated with LPS (0.1 µg/mL) only (Figure 5). The data suggest that diospyrin modulates the inflammatory reaction in LPS-induced mouse macrophages via p38 MAPK pathway.

## 4. Discussion

Various bioactivities of diospyrin such as anti-cancer and anti-leishmanial effects have been reported [5]. However, the effects of diospyrin on LPS-induced macrophages have yet to be fully reported.

Macrophages and monocytes are important mediators of innate reaction and inflammation against pathogenic bacterial infection [6,7,8,9,10]. Ferret et al. reported that macrophages and their circulating form monocytes mediate innate immunity and inflammatory reactions by presenting foreign antigens and scavenging dead cells [11]. Medina et al. reported that macrophages produce various inflammatory mediators such as cytokines and NO in the immune activity [12].

LPS, the main component of outer membrane of Gram-negative bacteria, has been shown to induce infection, inflammation, or tissue damage, as well as mediate inflammatory responses via MAPK pathway such as p38 MAPK and extracellular signal-regulated protein kinases (ERK) [13].

Lechner et al. reported that NO plays an important role in numerous physiological and pathophysiological conditions [14]. Thiemermann et al. reported that the expression of inducible nitric-oxide synthase and the production of large quantities of NO may contribute to the pathophysiology of endotoxemia or sepsis [15]. Evans et al. reported that excessive production of NO may be responsible, at least in part, for the hypotension associated with septic shock [16]. The current data show that diospyrin exerts inhibitory effects on the production of NO in LPS-induced RAW 264.7. Thus, diospyrin might be a candidate for modulating septic shock.

Kankkunen et al. reported that inflammasome is an intracellular molecular mediator of innate immunity in inflammation [17]. Martin et al. reported that inflammasomes are multiprotein complexes that activate caspase-1 in response to infections and stress, resulting in the secretion of pro-inflammatory cytokines [18], doi et al. reported that the activated macrophages produce pro-inflammatory cytokines such as MCP-1 [19]. Pavel et al. reported that cytokines regulate the immune response to infection by binding to cytokine receptors on the plasma membrane [20]. Gadient and Patterson reported that, in addition to the systemic acute phase reaction, IL-6 and LIF are associated with several acute and chronic inflammatory diseases, including rheumatoid arthritis and bacterial meningitis [21]. Ruddy et al. reported that the levels of various cytokines such as MCP-1, G-CSF, GM-CSF, and IP-10 are increased in bronchoalveolar fluid and the lung tissue of pneumococcal pneumonia during lung inflammation [22]. Capelli et al. reported that MCP-1 and MIP-1β levels are significantly increased in patients with chronic bronchitis [23]. Zhu et al. reported that the marked upregulation of RANTES in the epithelium and subepithelium exacerbates bronchitis [24]. Coussens and Werb reported that the tumor microenvironment, which is largely orchestrated by inflammatory cells, plays an indispensable role in the neoplastic transformation, proliferation, survival, and migration of cancer cells [25]. Kyama et al. have already reported that IL-6 and VEGF induce the development of endometriosis via excessive endometrial angiogenesis [26]. Appelmann et al. have also reported that VEGF overexpression has been linked to different types of malignancies and tumors [27]. Meanwhile, Dace et al. reported that IL-10, although traditionally considered as an anti-inflammatory cytokine, also contributes to the pathobiology of autoimmune diseases [28]. Meanwhile it could be recommended to investigate levels of cytokines simultaneously using pathogen-like molecules because cytokines are various. In this study, the data represent that diospyrin moderates excessive productions of inflammatory mediators such as MCP-1, MIP-1β, IL-6, IL-10, G-CSF, GM-CSF, VEGF, LIF, and RANTES in LPS-induced RAW 264.7. This means that diospyrin could be applied to treat bacterial infectious diseases such bacterial pneumonia and bacterial meningitis. In the addition, the current results mean that diospyrin might be effective to alleviate cytokine storm caused by bacterial infection. Cytokine storm is regarded as a serious and uncontrolled increase of cytokines’ level in the blood of infectious patients. Till now, treatments for cytokine storm are insufficient. Thus, diospyrin could be one of candidates for treating the uncontrolled cytokine storm revoked by bacterial infections.

Stout et al. reported that the ER calcium stores are reduced and intracellular calcium concentration is increased initially during the inflammation cascade [29]. Tabas et al. reported that the death receptor Fas is activated by CHOP, which amplifies the release of calcium from ER stores into the cytosol in ER-stressed macrophage [30]. Interestingly, Wang and Ron reported that CHOP was activated by p38 MAPK in the stressed cells [31]. Endo et al. reported that LPS triggers ER stress via overexpression of CHOP and activation of p38 MAPK, which mediates apoptosis in macrophages [32]. It is not easy to establish the MAPK signaling pathway including intracellular calcium release in inflammatory cascade. We focused on the activation of CHOP in RAW 264.7 stimulated by LPS, because CHOP is known to mediate the calcium–Fas–MAPK pathway. In the present study, diospyrin significantly inhibits the production of inflammatory cytokines, calcium release, and mRNA expression of CHOP and Fas in LPS-induced RAW 264.7 cells. Additionally, diospyrin significantly inhibited the phosphorylation of p38 MAPK in LPS-induced RAW 264.7 cells. These data suggest that diospyrin modulates LPS-induced macrophage activation via the ER-stressed calcium-p38 MAPK/CHOP/Fas pathway.

## 5. Conclusions

The present study demonstrates the anti-inflammatory effects of diospyrin via inhibition of NO, MCP-1, MIP-1β, IL-6, IL-10, G-CSF, GM-CSF, VEGF, LIF, and RANTES in LPS-induced macrophages mediated via the ER-stressed calcium-p38 MAPK/CHOP/Fas pathway. Further studies are needed to evaluate the medicinal benefits of diospyrin in inflammatory diseases.

## Figures and Tables

**Table 1:** Primers used for RT-PCR analysis.

Name ^1^	Forward Primer (5′–3′)	Reverse Primer (5′–3′)
*CHOP*	CCACCACACCTGAAAGCAG	TCCTCATACCAGGCTTCCA
*FAS*	CGCTGTTTTCCCTTGCTG	CCTTGAGTATGAACTCTTAACTGTGAG
*β-actin*	CTAAGGCCAACCGTGAAAAG	ACCAGAGGCATACAGGGACA

^1^ Primers’ names; C/EBP homologous protein (CHOP), first apoptosis signal receptor (FAS), β-actin.
